# Establishment of a Prognostic Nomogram for Patients With Locoregionally Advanced Nasopharyngeal Carcinoma Incorporating TNM Stage, Post-Induction Chemotherapy Tumor Volume and Epstein-Barr Virus DNA Load

**DOI:** 10.3389/fonc.2021.683475

**Published:** 2021-06-16

**Authors:** Yu-Ting Jiang, Kai-Hua Chen, Jie Yang, Zhong-Guo Liang, Song Qu, Ling Li, Xiao-Dong Zhu

**Affiliations:** ^1^ Department of Radiation Oncology, Guangxi Medical University Cancer Hospital, Nanning, China; ^2^ Department of Oncology, Affiliated Wuming Hospital of Guangxi Medical University, Nanning, China

**Keywords:** locoregionally advanced nasopharyngeal carcinoma, tumor volume, Epstein−Barr virus, nomogram, survival, prognosis

## Abstract

**Objectives:**

To establish and validate an effective nomogram to predict clinical outcomes for patients with locoregionally advanced nasopharyngeal carcinoma (LA-NPC).

**Materials and Methods:**

The clinicopathological parameters and follow-up information of 402 locoregionally advanced NPC patients (training cohort, n = 302; validation cohort, n = 100) were retrospectively enrolled. The nomogram was built with the important prognostic variables identified by Cox regression analysis. Overall survival (OS) and progression-free survival (PFS) were the primary and secondary endpoints, respectively. The predictive power and clinical utility of the nomogram were assessed using the Harrell concordance index (C-index), calibration curve, and decision curve analysis. We compared the eighth staging system model with the nomogram to analyze whether the model could improve the accuracy of prognosis

**Results:**

Epstein–Barr virus (EBV) DNA load, the gross tumor volume (GTVnx), and cervical lymph node tumor volume (GTVnd) after induction chemotherapy were the independent predictors of OS and PFS. The calibration curves indicated superb agreement between the nomogram-predicted probabilities and observed actual probabilities of survival. The C-index and area under the receiver operator characteristic curve (AUC) of the nomogram integrating these significant factors and N stage, and TNM stage were higher than those of the eighth TNM system alone. In addition, the decision curve analyses demonstrated the clinical value and higher overall net benefit of the nomogram. High-risk groups identified by the nomogram had significantly poorer OS and PFS than the low-risk group (p < 0.05).

**Conclusions:**

The multidimensional nomogram incorporating TNM stage, EBV DNA load, and tumor volume after induction chemotherapy led to a more precise prognostic prediction and could be helpful for stratifying risk and guiding treatment decisions in locoregionally advanced NPC patients who have undergone induction chemotherapy and concurrent chemoradiation.

## Introduction

Nasopharyngeal carcinoma (NPC) is a malignant tumor with a high incidence in South China. The majority of patients are defined as locoregionally advanced disease (LA-NPC, stages III-IVa) (1, 2). Concurrent chemoradiation (CCRT) is regarded as a standard treatment modality for patients with LA-NPC (3). In recent years, the addition of induction chemotherapy (IC) to the previously established regimen has received considerable attention. A previous randomized phase 2 study reported that the application of IC to CCRT was superior to CCRT alone for 3-year overall survival (OS, 94.1% *vs*. 67.7%, P = 0.012), and also led to a trend to improve progression-free survival (4). Later a phase 3 randomized controlled trial provided evidence that cisplatin/fluorouracil/docetaxel (TPF) induction chemotherapy (IC) followed by CCRT provided 5-year overall survival (OS: 85.6% *vs*. 77.7%, p = 0.042) benefits compared with CCRT alone in LA-NPC (5), and also improved long-term failure-free survival (FFS, 77.4% *vs*. 66.4%, p = 0.019), distant failure-free survival (DMFS, 88% *vs*. 79.8%, p = 0.030), and locoregional failure-free survival (FFS, 90.7% *vs*. 83.8%, p = 0.044). Other studies also indicated that TPF, cisplatin/fluorouracil (PF), or gemcitabine/cisplatin (GP) IC plus CCRT significantly improved tumor control and survival than CCRT alone (6–9). IC has since played an important role of the treatment regimen for LA-NPC. Although IC produces a survival benefit, 20% to 30% of patients continue to have local recurrence or distant metastasis after standard CCRT (10). Hence, developing a prognostic model for predicting survival outcome and early progression to guide risk stratification and treatment regimen modification is urgent.

Currently, the Union Internationale Contre le Cancer/American Joint Committee Cancer (UICC/AJCC) TNM staging system is widely applied for survival outcome prediction and treatment decision guidance in NPC. Nevertheless, the prognosis differs among patients with the same stage (11). The probable reason for this phenomenon is that the TNM staging system is mainly based on anatomic location, which is unable to reflect the biological variability of the tumor itself. Thus, we need a new effective prognostic model to refine the current TNM staging system and accurately predict which patients would benefit from more intensive treatment.

In this study, we used Cox regression analysis to identify important predictors and to develop a risk model for the prediction of 3-year OS and PFS probabilities and risk stratification in NPC patients.

## Materials and Methods

### Patients

Between January 2016 and June 2018, a total of 402 continuous patients in the Affiliated Cancer Hospital of Guangxi Medical University were enrolled in this retrospective study. The major inclusion criteria for this study were patients with biopsy-proven NPC and stages III-IVa disease, who were restaged according to the AJCC/UICC eighth edition. The other inclusion criteria included: [1] receiving IC with CCRT; [2] no history of radiotherapy, and/or chemotherapy before our study; and [3] clinical data, examination information, and follow-up data were available; and [4] no serious diseases or other sources of tumors when diagnosed with NPC. According to random numbers, 302 patients and 100 patients were randomly allocated into the training cohort and the validation cohort, respectively. The need to obtain informed consent was not required as this was a retrospective study.

### Chemotherapy and Radiation Therapy

All patients received platinum-based induction chemotherapy (IC) plus concurrent chemoradiation (CCRT) based on the institutional guideline recommendations. The regimens of IC included the TPF regimen (docetaxel + cisplatin + 5-fluorouracil, 60, 60, and 3,000 mg/m^2^, respectively), TP regimen (docetaxel + cisplatin, 75 and 75 mg/m^2^, respectively), GP regimen (gemcitabine + cisplatin, 1,000 and 80 mg/m^2^, respectively), or PF regimen (cisplatin+ 5-fluorouracil, 80 and 4,000 mg/m^2^, respectively) every 3 weeks for at least one cycle before CCRT. CCRT consisted of 3-weekly cisplatin (80–100 mg/m^2^) for two to three cycles.

Target volumes were outlined on the planning system by two radiation oncologists specializing in NPC according to our institutional treatment protocol. All patients received radical IMRT. The primary gross tumor volume (GTVnx) and cervical lymph node tumor volume (GTVnd) included the entire macroscopic tumor defined with the aid of computed tomography (CT), magnetic resonance imaging (MRI) scans, and physical examinations. For patients who have underwent 18F-fluorodeoxyglucose positron emission tomography with computed tomography (PET-CT), PET images were also used as a reference for target volumes delineation. Two clinical target volumes (CTVs) were delineated according to the tumor invasion pattern. The high-risk clinical target volume (CTV1) included the GTVnx add a margin of 0.5 to 1 cm (forward, both sides, up and down) and a margin of 0.3 to 0.5 cm (back) to encompass the high-risk sites of microscopic extension and the whole nasopharynx. The low-risk clinical target volume (CTV2) was defined as the CTV1 add a margin of 0.5 to 1 cm (forward, both sides, up and down) and a margin of 0.3 to 0.5 cm (back) to encompass the low-risk sites of microscopic extension, the GTVnd, and elective neck area from level IB to V. The planning target volume (PTV) was created by adding 3 to 5 mm to the CTV. The prescribed radiation doses to the PTVs of GTVnx, GTVnd, CTV1, and CTV2 were 70 to 75.9 Gy/31 to 32 f, 60 to 73.6 Gy/30 to 32 f, 60 to 68 Gy/30 to 31 f, and 54~57.6 Gy/30 to 31 f, respectively (11).

### EBV DNA Quantification

The pretreatment EBV DNA load (pre-DNA) and EBV DNA load after IC (post-DNA) were measured by real-time quantitative polymerase chain reaction (PCR) technique amplifying the BamHI-W fragment region of the EBV genome before and after IC as described previously (12). The results are shown as the number of copies of the EBV genome per milliliter of plasma. The cutoff level for post-DNA was based on a detectable/undetectable cutoff (1000 copies/mL, which defined by the clinical laboratory in the Affiliated Cancer Hospital of Guangxi Medical University), whereas the cutoff value for pre-DNA was set at 7000 copies/ml, which was calculated by receiver operator characteristic (ROC) curve analysis.

### Measurement of Tumor Volume

All NPC patients were immobilized with a tailor-made thermoplastic cast from head to shoulders. Contrast-enhanced computed tomography (CT) simulation scans at the radiotherapy position were performed on all patients. The scope of each scan was performed with a thickness of 3 mm from the top of the head to 2 cm below the lower edge of the clavicle. The contrast-enhanced CT images were transmitted into the radiotherapy planning system. The post-IC GTVnx and GTVnd were delineated on each slice of planning CT images according to the post-IC MRI image and calculated automatically by the treatment planning system. Retropharyngeal lymph nodes were encompassed in the GTVnx, as the retropharyngeal lymph node and primary tumor are so close that the discrimination of these anatomical sites remains difficult (13–15). The GTVnd included metastatic cervical lymph nodes and nodes with necrosis or extracapsular spread and nodal extracapsular spread based on pretreatment MRI (16, 17).

### Follow-Up and Endpoint

All patients were regularly checked at least 3 months during the first 2 years after RT, at least 6 months for the next 3 years, and annually thereafter until death. The evaluation of patients included clinical examination, nasopharyngoscopy, and imaging. Our main endpoint was overall survival (OS), which was defined as the interval between the date of diagnosis and the date of death or last follow-up. The secondary endpoint was progression-free survival (PFS), which was defined as the interval between the date of diagnosis and the date of diagnosis of treatment failure, death, or the last follow-up visit.

### Statistical Analysis

Clinicopathological variables associated with progression risk were assessed according to basic theoretical knowledge, clinical significance, and predictors confirmed by previous studies (11, 18–20). Continuous variables were converted into categorical variables, which are presented as a whole number and proportion. The optimal thresholds for pre-DNA, GTVnx, and GTVnd were calculated by receiver operating characteristic (ROC) curve analysis. Other variables were grouped according to the findings reported in previous studies (age, pre-LDH, pre-HGB, and pre-ALB) (11, 19, 20).

The statistical analyses were conducted using SPSS (version 25.0), R software (version 3.6.3), and X-tile software (version 3.6.1). The univariate and multivariate analyses were carried out using the Cox proportional hazards model to select independent prognostic variables of survival by a backward stepwise algorithm. The hazard ratio (HR) and 95% confidence interval were recorded (21). The eighth TNM staging system was recognized as a necessary prognostic indicator of survival. The nomogram was established incorporating all independent and necessary prognostic factors (by the “rms” package in R).

The predictive value of the nomogram was assessed by the predictive accuracy and discriminative ability. The concordance index (C-index) and calibration plot of the nomogram for OS and PFS at 3 years were applied to evaluate the performance of the established nomogram. The C-index was calculated to compare the discrimination ability of the nomogram with the current eighth TNM staging system, which was calculated by using the “Hmisc” package and 1000 bootstrap resamples. We further assessed the discriminative ability of the nomogram and the current TNM system using time-dependent receiver operating characteristic curve (tdROC) analysis with the “timeROC” package in R software. The area under the time-dependent ROC curve (tdAUC) was calculated to evaluate the sensitivity and specificity of models. In addition, decision curve analysis (DCA) was applied to evaluate the clinical usefulness of the models.

Finally, based on the optimal cutoff value of the risk score, which was determined by X-tile software, all patients were divided into high-risk and low-risk subgroups. Kaplan-Meier curves were used to compare the survival in the different risk subgroups in the training and validation cohorts with the log rank test (by the “survival” package in R).

P-values < 0.05 based on two-sided tests were considered to be statistically significant.

## Results

### Patient Characteristics

A total of 402 cases were enrolled for the analyses. An expected ratio of 1:3 was used for the training cohort (n = 302) and the validation cohort (n = 100) with random number. The characteristics and follow-up outcomes of the patients are shown in **Table 1**. There was no significant difference between the two cohorts except for the pre-DNA (p = 0.025). The median follow-up period was 45 (range, 5 to 85) months. The 1-, 3-, and 5-year overall survival (OS) and progression-free survival (PFS) rates of the entire cohort were 98.0%, 85.3%, 74.6% and 94.0%, 77.3%, 67.1%, respectively. In the training cohort, the median follow-up duration was 44 (range, 5–85) months, and the 1-, 3-, and 5-year OS and PFS rates were 98.0%, 84.6%, 73.0% and 93.4%, 75.9%, 64.3%, respectively, respectively. During the follow-up period, there were 65 deaths, and 64 patients had experienced distant metastasis. The median follow-up duration in the validation cohort was 46 (range, 5–84) months, and the 1-, 3-, and 5-year OS and PFS rates were 90.8%, 87.6%, and 79.2% and 94.9%, 80.4%, and 75.2%, respectively. By the final follow-up, 17 patients died, and 18 patients experienced recurrence and/or distant metastasis.

**Table 1:** Baseline characteristics of the patients. (n= 402).

Characteristics	Number of NPC patients (%)		
	Training cohort(n = 302)	Validation cohort(n = 100)	p-value
**Gender**			0.975
Male	223 (73.8)	74 (74)	
Female	79 (26.2)	26 (26)	
**Age (years)**			0.521
<50	161 (53.3)	57 (57)	
≥50	141 (46.7)	43 (43)	
**Smoking**			0.986
Yes	106 (35.1)	35 (35)	
No	196 (64.9)	65 (65)	
**T stage**			0.886
T1	1 (0.3)	0 (0)	
T2	77 (25.5)	23 (23)	
T3	105 (34.8)	35 (35)	
T4	119 (39.4)	42 (42)	
**N stage**			0.262
N0	11 (3.6)	2 (2)	
N1	89 (29.5)	27 (27)	
N2	116 (38.4)	50 (50)	
N3	86 (28.5)	23 (23)	
**TNM stage**			0.724
III	117 (38.7)	41 (41)	
IVA	185 (61.3)	59 (59)	
**WHO histological type**			0.713
WHO I	2 (0.7)	0 (0)	
WHO II	28 (9.3)	9 (9)	
WHO III	272 (90)	91 (91)	
**pre-Hb (g/L)**			0.502
<120	39 (12.9)	16 (16)	
≥120	263 (87.1)	84 (84)	
**pre-ALB (g/L)**			0.376
<45	265 (87.7)	91 (91)	
≥45	37 (12.3)	9 (9)	
**pre-LDH (IU/L)**			0.357
<180	164 (54.3)	49 (49)	
≥180	138 (45.7)	51 (51)	
**pre-DNA (copies/ml)**			**0.025**
<7000	198 (65.6)	53 (53)	
≥7000	104 (34.4)	47 (47)	
**post-DNA**			0.964
Undetectable	216 (71.5)	72 (72)	
Detectable	86 (28.5)	29 (29)	
**GTVnx (cc)**			0.487
<90	184 (60.9)	57 (57)	
≥90	118 (39.1)	43 (43)	
**GTVnd (cc)**			0.373
<30	127 (42.1)	37 (37)	
≥30	175 (57.9)	63 (63)	
**Clinical endpoints**			0.468
None	210 (69.5)	78 (78)	
Recurrence	21 (7)	4 (4)	
Distant metastasis	40 (13.2)	14 (14)	
Recurrence and distant metastasis	3 (1)	3 (1)	
Death	65 (21.5)	17 (17)	

Data are shown as numbers (%). WHO, World Health Organization; Hb, hemoglobin; ALB, serum albumin; LDH, lactate dehydrogenase; EBV, Epstein-Barr virus; GTVnx, primary gross tumor volume; GTVnd, cervical lymph node tumor volume; cc, cubic centimeter. Bolded values: p < 0.05.

### Prognostic Factors

According to the univariate analysis, patients with higher pre-DNA (≥7000 copies/ml), post-DNA (detectable), and pre-LDH (≥180 U/L), larger GTVnx (≥ 90 cc) and GTVnd (≥ 30 cc), and advanced N stage and AJCC/UICC eighth TNM stage were related to poorer OS and PFS (all p < 0.05, **Table 2**). We used Spearman correlation analysis to decrease the degree of multicollinearity and found the correlations between N stage and GTVnd, and TNM stage and post-DNA were both significant (p < 0.001 and p = 0.03, respectively). Thus, it was not reasonable to include these factors in the multivariate analysis at the same time. N stage and TNM stage are recognized as necessary indicators for predicting survival, so they were not incorporated into the multivariate analysis and were added to the nomogram directly. In the multivariate analysis (**Table 3**), post-DNA, GTVnx, and GTVnd remained independent predictor and were selected as the significant prognostic variables together with N stage and TNM stage for inclusion in the nomogram. The visual details of the univariate and multivariate analyses associated with OS and PFS are shown in **Tables 2** and **3**.

**Table 2:** Identification of risk factors of OS and PFS by univariate Cox models.

Variable	OS		PFS	
	HR (95% CI)	P-value	HR (95% CI)	P-value
**Gender**		0.157		0.459
Female	Reference		Reference	
Male	1.551 (0.844–2.851)		1.199 (0.742–1.936)	
**Age (years)**		0.488		0.478
<50	Reference		Reference	
≥50	1.192 (0.726–1.958)		1.162 (0.767–1.759)	
**Smoking status**		0.685		0.799
No	Reference		Reference	
Yes	1.111 (0.668–1.849)		1.063 (0.692–1.633)	
**T stage**		0.605		0.549
T1/2	Reference		Reference	
T3	0.808 (0.416–1.568)		0.881 (0.505–1.537)	
T4	1.082 (0.596–1.963)		1.152 (0.692–1.915)	
**N stage**		**<0.001**		**<0.001**
N0/1	Reference		Reference	
N2	2.050 (1.029–4.086)		1.811 (1.047–3.133)	
N3	3.426 (1.938–7.571)		3.033 (1.744–5.276)	
**TNM stage**		**0.013**		**0.008**
III	Reference		Reference	
IVA	2.051 (1.166–3.607)		1.870 (1.181–2.963)	
**pre-Hb (g/L)**		0.926		0.466
<120	Reference		Reference	
≥120	1.036 (0.494–2.171)		1.277 (0.662–2.462)	
**pre-ALB (g/L)**		0.255		0.593
<45	Reference		Reference	
≥45	1.443 (0.768–2.713)		1.170 (0.658–2.079)	
**pre-LDH (IU/L)**		**0.026**		**0.027**
<180	Reference		Reference	
≥180	1.752 (1.069–2.870)		1.591 (1.054–2.401)	
**pre-DNA (copies/ml)**		**0.007**		**0.001**
<7000	Reference		Reference	
≥7000	1.960 (1.202–3.195)		2.040 (1.354–3.072)	
**post- DNA**		**<0.001**		**<0.001**
Undetectable	Reference		Reference	
Detectable	2.527 (1.548–4.126)		2.132 (1.412–3.219)	
**GTVnx (cc)**		**0.044**		0.093
<90	Reference		Reference	
≥90	1.649 (1.014–2.684)		1.422 (0.943–2.144)	
**GTVnd (cc)**		**<0.001**		**0.001**
<30	Reference		Reference	
≥30	3.374 (1.862–6.113)		3.374 (1.862–6.113)	

Hb, hemoglobin; ALB, serum albumin; LDH, lactate dehydrogenase; EBV, Epstein-Barr virus; GTVnx, primary gross tumor volume; GTVnd, cervical lymph node tumor volume; cc, cubic centimeter. Bolded values: p < 0.05.

**Table 3:** Identification of risk factors of OS and PFS by multivariate Cox models.

Characteristics	OS		PFS	
	HR (95% CI)	P-value	HR (95% CI)	P-value
**LDH (IU/L)**		0.277		0.284
<180	Reference		Reference	
≥180	1.334 (0.793–2.244)		1.269 (0.821–1.962)	
**pre-DNA (copies/ml)**		0.455		0.099
<7000	Reference		Reference	
≥7000	1.245 (0.701–2.213)		1.496 (0.927–2.413)	
**post-DNA**		**0.003**		**0.042**
Undetectable	Reference		Reference	
Detectable	2.136 (1.298–3.517)		1.645 (1.017–2.660)	
**GTVnx (cc)**		**0.049**		0.093
<90	Reference		Reference	
≥90	1.648 (1.003–2.709)		1.422 (0.943–2.144)	
**GTVnd (cc)**		**<0.001**		**0.002**
<30	Reference		Reference	
≥30				

LDH, lactate dehydrogenase; EBV, Epstein-Barr virus; GTVnx, primary gross tumor volume; GTVnd, cervical lymph node tumor volume; cc, cubic centimeter. Bolded values: p < 0.05.

### Nomogram Development and Validation

**Figure 1:**
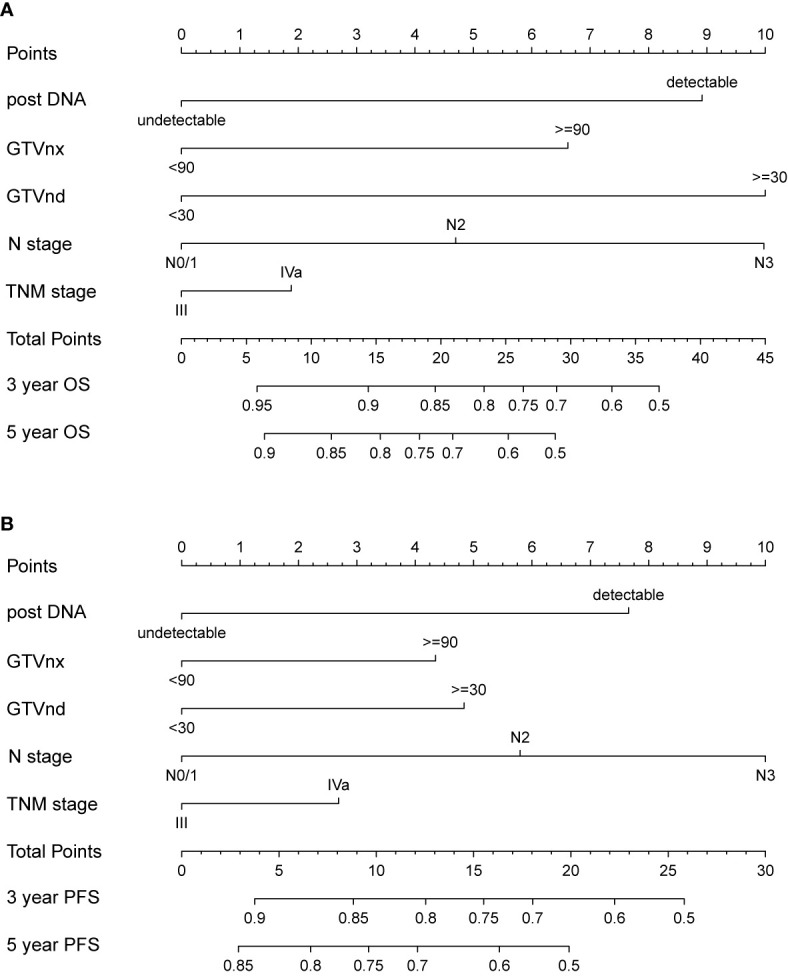
Nomogram integrating N stage, TNM stage and all independent clinical factors (post-DNA, GTVnx, and GTVnd) for predicting 3- and 5-year OS **(A)** and PFS **(B)**.

**Figure 2:**
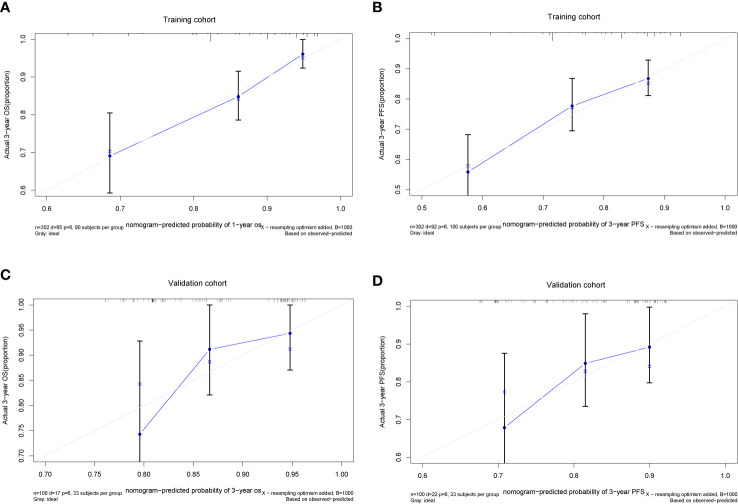
The Calibration plots of the nomogram in predicting OS and PFS at 3 year in the training **(A, B)** and validation cohorts **(C, D)**.

We further established two nomograms for OS and PFS that integrated all potential significant prognosticators in the training group (**Figures 1A, B**). We can add the score of each variable up for the total score and locate it on the survival rate scale to predict 3- and 5- year OS and PFS. The C-indexes of the nomogram for OS and PFS were 0.716 (95% CI, 0.652–0.780) and 0.680 (95% CI, 0.622–0.738), respectively. According to the calibration plots, the actual 3-year OS or PFS rate is plotted on the y-axis, and the x-axis represents the nomogram-predicted probability of 3-year OS or PFS, respectively. As shown in **Figures 2A, B**, the calibration plot exhibited a remarkable concordance between the actual value and prediction in the training cohort.

The C-index and calibration slope were applied to validate the nomogram accuracy in the validation cohort. The Harrell C index for predicting OS and PFS were 0.676 (95% CI, 0.550–0.709) and 0.629 (95% CI, 0.512–0.746), respectively. As shown in **Figures 2C, D**, the calibration curves for the validation cohort also showed a superb calibration between actual-observed and nomogram-estimated OS or PFS, which indicated that the nomogram was well calibrated.

### Comparison Between the Eighth UICC/AJCC TNM Staging System and the Nomogram in Prediction Accuracy

**Figure 3:**
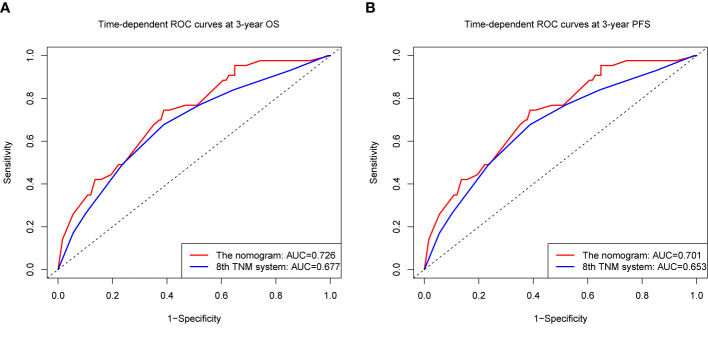
The time-dependent receiver operating characteristic (tdROC) curves of OS **(A)** and PFS **(B)** at 3 year by the nomogram and the eighth UICC/AJCC TNM staging system.

**Figure 4:**
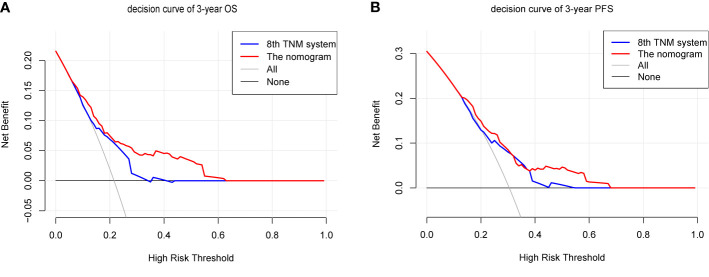
The decision curves (DCA) of OS **(A)** and PFS **(B)** at 3 year by the nomogram and the eighth UICC/AJCC TNM staging system.

We compared the accuracy in predicting survival of the eighth TNM staging system and the nomogram in the training cohort to find a model with the highest predictive value. The C-index of OS and PFS for the TNM staging system were 0.663 (95% CI, 0.595–0.731) and 0.641 (95% CI, 0.581–0.701), respectively, which were smaller than the C-index of the nomogram. It revealed that the nomogram produced a consistently significant improvement over the eighth TNM staging system in predicting OS and PFS. The time-dependent ROC (tdROC) curves of 3-year OS and PFS demonstrated that the nomogram (3-year OS: AUC = 0.726; 3-year PFS: AUC = 0.701) represented a more feasible model than the eighth TNM staging system (3-year OS: AUC = 0.677; 3-year PFS: AUC = 0.653) (**Figures 3A, B**). Furthermore, the decision curve analysis indicated that the clinical application of the nomogram in predicting OS and PFS had a higher net benefit than the eighth TNM staging system (**Figures 4A, B**). Additionally, we calculated the NRI to determine whether the nomogram with the new factors refined the prognostic ability of the current TNM staging system. The results of NRI for OS and PFS were 0.098 (95% CI: −0.056 to 0.441) and 0.064 (95% CI: −0.086 to 0.386), respectively, which suggests that the new model had more robust predictive power.

### Recognition of Low- and High-Risk Groups by the Nomogram

**Figure 5:**
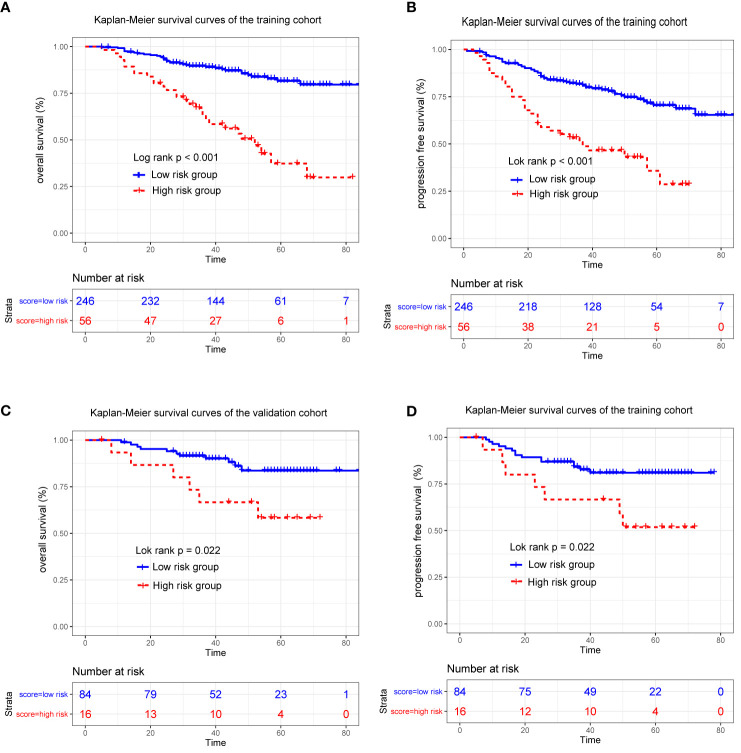
Kaplan-Meier survival curves for patients stratified based on the nomogram in the training **(A, B)** and validation cohort **(C, D)**.

Because the nomogram had better predictive ability than the eighth edition TNM staging system, stratification of the data was conducted based on the optimal cutoff point of the total linear prediction score (by X-tile software) derived from the nomogram. All patients were separated into two risk groups: a high-risk group (total score ≥28) and a low-risk group (total score <28). Obviously, the Kaplan–Meier survival curve showed that OS and PFS of patients in the low-risk group were substantially increased compared to patients in the high-risk group in the training cohort (3-year OS 62.8 *vs* 89.6%, 3-year PFS 51.0% *vs* 81.7%, p < 0.001) (**Figures 5A, B**) and validation cohort (3-year OS 66.7% *vs* 91.5% and 3-year PFS 66.7% *vs* 84.2%, p < 0.05) (**Figures 5C, D**), which further verified that our nomogram could effectively stratify patients at risk in this study.

## Discussion

In this study, a nomogram was developed and validated by a retrospective analysis of patients with locoregionally advanced NPC to predict OS and PFS individually and showed good prognostic value in clinical decision making. The results showed that tumor volume and EBV DNA load after induction chemotherapy (IC) are independent predictors for survival. Our nomogram was established by the addition of post-IC tumor volume and EBV DNA load to the eighth UICC/AJCC TNM staging system and improved prognostic accuracy, which would assist clinicians in guiding further risk stratification and early treatment modification.

In endemic areas, NPC is an Epstein-Barr virus (EBV)-driven malignancy. Previous studies have reported that pretreatment EBV DNA load is considered to be an indicator of tumor load, and is correlated with risk stratification and prognosis prediction (22–25). In addition, Li’s (19) and Yang’s (18) studies both developed a nomogram for survival prediction using pretreatment EBV-DNA load. However, the clinical prognostic models containing EBV DNA load after IC is limited. Some studies demonstrated that the post-DNA is a powerful and early prognostic factor in LA-NPC patients (26, 27). Our results agree with the findings of these studies, which found that detectable post-DNA is an effective factor in predicting treatment failure. The mechanism behind this phenomenon may be related to the relatively unsatisfactory sensitivity of chemotherapy in patients with detectable post-DNA, and it reflects the existence of residual tumor cells. More specifically, EBV DNA may originate from necrotic or apoptotic cells and is representative of tumor DNA levels (28).

In clinical practice, treatment decisions mainly depend on the current TNM staging system, which does not accurately reflect the tumor burden. Tumor volume represents the tumor burden and can be easily calculated from the radiotherapy planning system. The Response Evaluation Criteria in Solid Tumors criteria mainly measure the reduction or enlargement in the maximum diameter of the tumor on the two-dimensional imaging (29). However, the value of using traditional response evaluation criteria to evaluate the response of NPC patients to IC is limited in clinical practice, as the primary tumor of LA-NPC patients is irregular in shape and tends to invade the bone structure at the base of the skull. Therefore, it may not be appropriate to evaluate the response based on the conventional criteria for LA-NPC. In contrast, the tumor volume provides three-dimensional information that more intuitively and accurately reflects the response to treatment, thus reflecting the patient’s sensitivity to chemotherapy (30). Multiple studies have suggested that tumor volume is significantly associated with survival outcome (31–33). However, a major number of studies have focused on the prognostic significance of pretreatment tumor volume (34, 35), and the potential of post-IC tumor volume for the prediction of prognosis and guidance of treatment regimens remains unclear. Chen et al. (36) found that post-IC GTVnx and GTVnd are considered indicators of survival prognosis and have important significance in guiding treatment decisions. Intriguingly, according to our findings, we found that a larger post-IC GTVnx or GTVnd was related to a significantly worse clinical outcome compared than a smaller post-IC GTVnx or GTVnd for 3-year OS and PFS. Hence, post-IC tumor volume can not only reflect tumor load but also represent the chemotherapeutic sensitivity of IC to a certain extent and can guide the risk stratification of LA-NPC.

Based on the conclusions of these studies, the levels of post-DNA, GTVnx, and GTVnd were evaluated in this study and used as independent prognostic variables to establish the nomograms. The TNM staging system is based primarily on anatomical information, and it lacks precision in stratifying patients at risk and individual therapy. The prognostic nomograms of OS and PFS were established in combination with the important prognostic factors in this study, and the C index and AUC were higher than those of the eighth TNM staging system, which indicated that our models were better than the current staging system in terms of prognostic efficiency. Recently, the net reclassification improvement (NRI) has been recognized as a new indicator that can be used to compare the accuracy of the predictive power of the two models. We used the NRI to quantify the improvement in survival prediction from the addition of some new markers compared to the traditional staging. Adding post-IC EBV and tumor volume on the basis of the traditional TNM staging system yielded NRI of 0.098 for OS and 0.064 for PFS. In addition, patients can be divided into different risk subgroups according to the total score produced by the nomogram, in which the patients in the high-risk group had a poorer prognosis. Our results demonstrated that the new model could improve the predictive power and the accuracy of individual risk stratification of the eighth TNM staging system.

In the era of rapid development of modern treatment, individualized treatment becomes particularly important. Patients in the high-risk group are likely to have a higher tumor burden. Thus, identifying high-risk patients as early as possible may be the key to improve survival outcomes and will assist in guiding treatment decisions prior to CCRT. The nomogram in our study shows great potential for application in clinical practice. Clinicians could evaluate the condition of LA-NPC patients before the implement of CCRT using the model and select high-risk patients who may benefit from intensive treatment. Specifically intensive treatment after IC included the following: [1] increase the cumulative cisplatin dose during CCRT. Many patients who receive IC before CCRT experience grade 3 or 4 adverse events, which would reduce their tolerance to concurrent chemotherapy (7). Therefore, it is important to screen patients with LA-NPC who may benefit from a higher cumulative cisplatin dose (CCD). Wen et al. demonstrated that high-risk patients with advanced T or N stage, higher pre-DNA or larger post-IC tumor volume benefited from CCD ≥ 200 mg/m^2^ for PFS and DMFS (37). A previous study also showed that patients with detectable post-DNA showed significantly improved 3-year PFS and LRFS by receiving ≥ 160 mg/m^2^ CCD (38). [2] Add targeted therapy to the treatment regimen. Epidermal growth factor receptor (EGFR) is also highly expressed in NPC (39) and a previous study has shown that the application of cetuximab (CTX) or nimotuzumab (NTZ) (anti-EGFR monoclonal antibodies) was a significant protective factor for OS, DFS, and DMFS in patients treated with CCRT (40). [3] Add in adjuvant chemotherapy to treatment. Previous studies have demonstrated a survival benefit from adjuvant chemotherapy in high-risk subgroups (such as advanced T and N stage, higher NLR and LDH) by stratifying patients for risk (11, 41, 42). However, it has also been shown that adding adjuvant chemotherapy to CCRT increases acute toxicity in patients with LA-NPC (43, 44). Therefore, the application of traditional intravenous adjuvant chemotherapy regimens in NPC is controversial. Previous retrospective studies have shown that oral fluorouracil chemotherapeutic agents (such as capecitabine) are well tolerated in adjuvant chemotherapy, can be protective factors for survival and may be more suitable for patients with radical radiotherapy (42, 45, 46). [4] Increase the use of immunotherapy. Some features of NPC justify the use of immunotherapy, such as the association with EBV infection, upregulation of PD-L1 expression, and a high number of tumor-infiltrating lymphocytes (47). For example, the discovery of the co-inhibitory molecules PD-1 and PD-L1 were groundbreaking event for immunotherapy in this era of personalized management. For patients with PD-L1-positive NPC, a 22% response rate was observed after anti-PD-1 treatment (48). [5] Increase the radiotherapy dose. Some studies have reported that the minimum point dose (Dmin) escalation to the primary gross tumor volume may lead to better local control (10, 49). Our findings indicated that although treatment failure in LA-NPC is not uncommon, low-risk patients may have a better prognosis with a relatively lower tumor burden. Intensive treatments, such as targeted therapy, immunotherapy, and adjuvant therapy, may not produce survival benefits but increase the risk of toxicity and the economic burden. Moreover, a previous study has confirmed that a higher cumulative cisplatin dose during CCRT was not superior to a lower one in terms of survival for LA-NPC patients in the low-risk group with a smaller post-IC tumor volume or undetectable post-IC EBV DNA (37, 38). As a result, for these patients, screening needs to be early and the intensity of treatment can be reduced to avoid unnecessary toxicities and costs.

The advantages of this study are as follows. First, this is the first study ever reported to establish a model that features independent variables after IC in LA-NPC patients. The results of this study have certain educational and practical relevance. Second, in most healthcare institutions, the variables involved in the nomogram are readily available, so the model has broad clinical applicability. Finally, the nomogram is a visually predictive tool that can be used in clinical practice to help doctors quickly assess the prognosis of patients through simple calculations, and to classify patients with different severities to help determine individualized treatment.

Our study had some limitations. First, our follow-up time was not long enough and the number of patients was relatively small because of the key inclusion criteria. There should be a longer follow-up time and a larger study population if possible. Second, studies from a single institution do not provide robust evidence; we need prospective studies with large cohorts to validate the conclusion. Moreover, radiomics is a new research hot topic that has been confirmed by an increasing number of research studies to have strong prognostic potential (18, 50). As the technology to acquire radiomic features of medical imaging has not been widely developed in our center, this study failed to discuss it in depth. The further study of radiomics is needed. Lastly, similar to other retrospective studies, the selection bias was unavoidable. However, this retrospective study has clinical significance because it provides a reference for the planning of some prospective studies.

In summary, our study developed and validated a novel model for predicting OS and PFS rates after induction chemotherapy for LA-NPC patients in an endemic area that involved the eighth TNM staging system, post-IC EBV DNA and tumor volume. The predictive value of the nomogram was obviously better than that of the current eighth TNM staging system. In the future, prospective studies are required to generalize the clinical usefulness of our model.

## Data Availability Statement

The original contributions presented in the study are included in the article/supplementary material. Further inquiries can be directed to the corresponding author.

## Ethics Statement

The studies involving human participants were reviewed and approved by the Ethics Committee of the Affiliated Cancer Hospital of Guangxi Medical University. The ethics committee waived the requirement of written informed consent for participation. Written informed consent was not obtained from the individual(s) for the publication of any potentially identifiable images or data included in this article.

## Author Contributions

Study concepts: X-DZ and Y-TJ. Study design: X-DZ, Y-TJ, and K-HC. Data acquisition: Y-TJ, K-HC, JY, and Z-GL. Quality control of data and algorithms: Y-TJ and K-HC. Data analysis and interpretation: Y-TJ, K-HC, JY, and Z-GL. Statistical analysis: Y-TJ, K-HC, SQ, and LL. Manuscript preparation: Y-TJ, K-HC, JY, and Z-GL. Manuscript editing: Y-TJ, K-HC, SQ, and LL. Manuscript review: SQ, LL, and X-DZ. All authors read and X-DZ approved the final manuscript. All authors contributed to the article and approved the submitted version.

## Funding

This work was supported by the Key Research and Development Program Project of Guangxi Zhuang Autonomous Region (grant GuikeAB18221007) and the Independent Project of Key Laboratory of Early Prevention & Treatment for Regional High-Incidence Tumor (grant GKE-ZZ202014).

## Conflict of Interest

The authors declare that the research was conducted in the absence of any commercial or financial relationships that could be construed as a potential conflict of interest.
